# Simple and Large Scale Construction of MoS_2_-g-C_3_N_4_ Heterostructures Using Mechanochemistry for High Performance Electrochemical Supercapacitor and Visible Light Photocatalytic Applications

**DOI:** 10.1038/srep43055

**Published:** 2017-02-27

**Authors:** Sajid Ali Ansari, Moo Hwan Cho

**Affiliations:** 1 School of Chemical Engineering, Yeungnam University, Gyeongsan-si, Gyeongbuk 712-749, South Korea

## Abstract

The design of heterojunctions for efficient electrochemical energy storage and environmental remediation are promising for future energy and environment applications. In this study, a molybdenum disulfide-graphitic carbon nitride (MoS_2_-g-C_3_N_4_) heterojunction was designed by applying simple mechanochemistry, which can be scaled up for mass production. The physical-chemical and photophysical properties of the as-prepared MoS_2_-g-C_3_N_4_ heterojunction were analyzed using a range of characterization techniques. The supercapacitance performance was determined by electrochemical half-cell measurements, and visible light-induced photoelectrochemical and photocatalytic performance was studied using photocurrent and model organic pollutant degradation experiments. The resulting MoS_2_-g-C_3_N_4_ under the optimized experimental conditions showed significantly higher photocatalytic activity and photoelectrochemical performance under similar visible photoirradiation conditions compared to the bare materials. The resulting heterostructure electrode delivered a higher capacitance of 240.85 F/g than the bare material (48.77 F/g) with good capacitance retention. The superior performance was attributed mainly to the robust light harvesting ability, improved charge separation, high surface area, increased mass transfer, and capacitive and conductive behavior. The convenient and mass production of heterojunctions using a simple and cost-effective method will provide a good example for the efficient design of visible light active photocatalysts and capacitor electrode materials for environmental remediation and energy storage device applications.

The development of heterostructured photocatalysts with high activity and the ability to utilize the maximum energy part of the solar spectrum has attracted considerable attention in the field of energy and the environment to solve various energy and environmental issues^1^,^2^,^3^,^4^,^5^. A range of semiconductors, particularly titanium dioxide (TiO_2_), have been reported to be photoactive catalysts for the degradation of organic pollutants, but their photocatalytic applications in the visible light region have been hindered by their wide band gap and high rate of photoinduced electron/hole recombination^1^,^2^,^3^,^4^,^5^. Therefore, exploring a new visible light photocatalysts that may be a more efficient alternative than TiO_2_ is a hot topic in visible light photocatalysis^1^,^2^,^3^,^4^,^5^.

The impressive characteristics of supercapacitors, such as the immediate delivery of a higher power density with simultaneously shorter charging times, have attracted considerable attention in portable electronic device applications. Owing to these characteristics, tremendous research efforts have been carried out to develop new supercapacitive electrode materials for environmental and energy applications with low cost but with a high power density because the electrode material plays an important role in the development of effective and high performance supercapacitor electrodes^6^. The capacitance of the electrode depends on the characteristics of the materials, such as conductivity, stability, and theoretical capacitance. Accordingly, there have been many developments on carbon-based supercapacitive electrode materials owing to their large surface area, good electronic conductivity, and lightweight but their performance has been unsatisfactory due to the insufficient penetration of ions on the inert surface. Therefore, to solve the abovementioned problems, nitrogen-rich, carbon-based materials have attracted increasing interest due to the presence of nitrogen, which improves the surface polarity, electron donor properties, electric conductivity, and surface wettability^7^,^8^,^9^.

Two dimensional graphite-like structures, such as graphitic-like carbon nitride (g-C_3_N_4_), have attracted considerable interest in the field of metal-free photocatalysts and supercapacitor electrode materials owing to its suitable band gap, special optical and physicochemical features, high nitrogen content, cadenced carbon and nitrogen framework, and easy synthesis process^10^,^11^,^12^,^13^,^14^,^15^,^16^. In addition, the presence of nitrogen in g-C_3_N_4_ itself plays an important role in improving the wettability of the electrode with the electrolytes, electron donor/acceptor, provides more active reaction sites, and produces large additional pseudo-capacitance behavior^6^,^7^,^17^. Nevertheless, similar to the other semiconductor photocatalyst and electrode material, bare g-C_3_N_4_ also exhibits an unsatisfactory light absorption response due to the low separation efficiency of the photoinduced electrons holes and the low surface area^1^,^2^.

Recently, two-dimensional molybdenum disulfide (MoS_2_) dichalcogenide, which is comprised of Mo metal layers sandwiched between two sulfur layers and stacked together by weak van der Waals interactions has attracted a great deal of attention in the field of photocatalytic and energy storage applications owing to its excellent electrocatalytic performance, strong absorption ability in the visible region, narrow band gap, good conductivity, high theoretical specific capacitance, and good cycling stability^18^,^19^. In addition, the unique band structure of MoS_2_ can form good energy band alignment with other two dimensional layered structures, which would help promote the transfer of photo-generated electrons and holes effectively. On the other hand, the poor electronic conductivity restricts the high capacitance performance, whereas its bulk structure shows moderate photocatalytic activity that limits its applications in energy storage and environmental remediation^20^,^21^,^22^,^23^. Many methods have been used to produce MoS_2_-g-C_3_N_4_ heterojunctions, which have their own advantages and disadvantages in terms of the use of excess chemicals, whose discharge may directly or indirectly affect the environment, and complicated synthetic steps. For example, Zhao *et al*.^14^ used a solution-based route for the synthesis of hybrid nanostructures, whereas Li *et al*.^16^ and Yan *et al*.^24^ used a solvothermal method to prepare MoS_2_-g-C_3_N_4_ heterostructures and tested their photocatalytic activity. Therefore, a facile method that can be used for large-scale production is needed. Simple mechanochemistry-based methods are considered to be a good energy-saving technology and a highly efficient method for the design of heterostructures because of its potential to strengthen the interfacial interaction between the materials, reduce the layered thickness of the layered materials, lower the activation energy, and improve the material performance.

In terms of the above discussion of the desirable properties of g-C_3_N_4_ and MoS_2_, it is important to design a rational heterojunction that would exploit the unique characteristics and physicochemical properties of both materials as well as to develop an effective and highly visible light driven photocatalyst and electrochemical supercapacitive electrode.

In this study, a facile and sustainable synthesis method was developed for the synthesis of large scale two dimensional molybdenum disulfide-graphitic carbon nitride (MoS_2_-g-C_3_N_4_) heterostructures for energy and environmental applications. The reported method is simple and relatively inexpensive, and can be scaled up for mass production. The resulting MoS_2_-g-C_3_N_4_ under the optimized experimental conditions exhibit higher photocatalytic, photoelectrochemical performance compared to the bare materials under similar visible photoirradiation. The electrochemical half-cell electrochemical supercapacitance of MoS_2_-g-C_3_N_4_ is reported. The results show that MoS_2_-g-C_3_N_4_ delivers higher electrochemical capacitance than the as-synthesized bare materials. The excellent performance was attributed mainly to the construction of unique heterojunctions, expanded visible light absorption ability, high surface area, charge storage nature, and conductive behavior. This study provides a new way to develop a mass level of the material for the heterogeneous catalysis in solar energy conversion and energy storage materials.

## Results and Discussion

### Structural and Photophysical Characteristics

**Figure 1:**
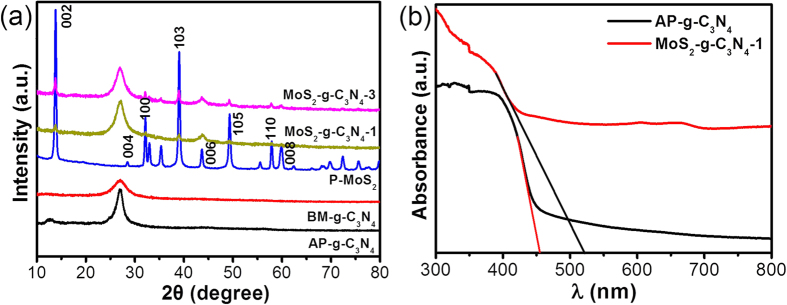
(**a**) XRD patterns of AP-g-C_3_N_4_, BM-g-C_3_N_4_, P-MoS_2_, MoS_2_-g-C_3_N_4_-1, and MoS_2_-g-C_3_N_4_-3 heterostructure and (**b**) UV-visible diffuse absorbance spectra of AP-g-C_3_N_4_ and MoS_2_-g-C_3_N_4_-1 heterostructure with direct band gap measurement.

The crystal phase and crystal structures of AP-g-C_3_N_4_, BM-g-C_3_N_4_, P-MoS_2_, MoS_2_-g-C_3_N_4_-1, and MoS_2_-g-C_3_N_4_-3 were examined by X-ray diffraction (XRD). The phase of all the photocatalysts was indexed to the specific planes, as marked in the Fig. 1a, which is in good agreement with the previous reported patterns^1^. AP-g-C_3_N_4_ and BM-g-C_3_N_4_ had a characteristic XRD peak at 27.47° 2θ, which was assigned to the tight interplanar stacking of the aromatic planes in g-C_3_N_4_ (Fig. 1a). The XRD pattern of BM-g-C_3_N_4_ displayed a broader peak compared to the AP-g-C_3_N_4_, which is due likely to the decrease in size and layer thickness. The XRD pattern of MoS_2_-g-C_3_N_4_ with different MoS_2_ contents clearly shows the characteristic peak for MoS_2_ in the present heterostructure, which confirmed the successful formation of the MoS_2_-g-C_3_N_4_ heterostructure during the high energy ball milling process^23^. The characteristic XRD peaks of MoS_2_ and g-C_3_N_4_ were visible in the XRD pattern of the MoS_2_-g-C_3_N_4_ heterostructure, which suggests that the content and reaction time are suitable for the successful formation of MoS_2_-g-C_3_N_4_ heterostructures. In addition, the XRD peak of MoS_2_ and g-C_3_N_4_ in the MoS_2_-g-C_3_N_4_ heterostructure was broader after mechanical milling. This might be due to the decrease in size and layer thickness of the heterostructure, which might be helpful in imparting novel and improved characteristics to the present heterostructures. Recently, many studies have shown that the mechanical grinding of layered structures by ball milling reduces the size and thickness of the layered materials significantly. For example, Zhu *et al*.^11^ reported that the catalytic performance of bulk g-C_3_N_4_ can be improved significantly by reducing the thickness of g-C_3_N_4_ simply by increasing the mechanical grinding time. Similarly, Song *et al*.^25^ and Krishnamoorthy *et al*.^20^ reported that simple mechanical grinding is a good approach to the synthesis of smaller size particles and layered materials with a few layers because of the high energy shear forces induced during the mechanical process^20^,^24^. Therefore, MoS_2_ and staked g-C_3_N_4_ with different lateral sizes and thicknesses are formed from their bulk form through mechanical grinding^11^.

The light absorption characteristics and electronic band structure of the MoS_2_-g-C_3_N_4_ heterostructure were investigated by UV-vis diffuse absorbance spectroscopy; the results are presented in Figs 1b and S1. The absorption edge of the MoS_2_-g-C_3_N_4_ heterostructure showed a red shift and slightly enhanced light absorption in the visible region compared to the bare g-C_3_N_4_. This enhanced absorption is in good agreement with the observed color change from light yellow to gray^25^. The band gap was calculated directly from the absorption spectra of g-C_3_N_4_ and MoS_2_-g-C_3_N_4_ heterostructure and found to be ~2.75 eV for g-C_3_N_4_, which can be attributed to the characteristic band gap of g-C_3_N_4_, and ~2.35 eV for the MoS_2_-g-C_3_N_4_ heterostructure. The shift of the absorption edge and the substantial decrease in the band gap may be due to the interaction and synergism between MoS_2_ and g-C_3_N_4_. These results show that the extended absorption in the visible-light region may be helpful in absorbing more visible light and producing a large number of charge carriers, which may be favorable for improving the photocatalytic and photoelectrochemical performance^14^,^24^.

**Figure 2:**
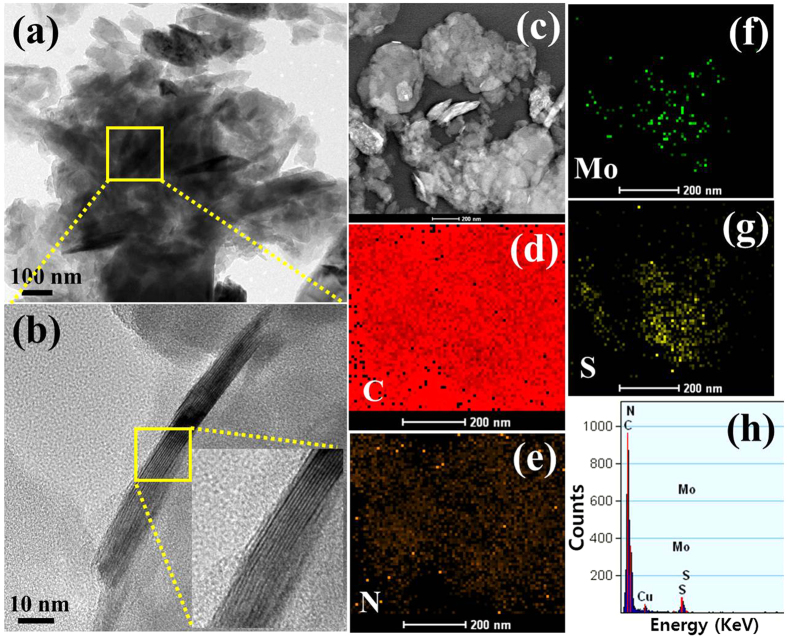
(**a** and **b**) TEM images of the MoS_2_-g-C_3_N_4_-1 at different magnification scale, (**c**–**g**) scanning transmission electron microscopy elemental mapping, and (**h**) EDX of the MoS_2_-g-C_3_N_4_-1.

The microstructure and morphology of the MoS_2_-g-C_3_N_4_-1 was examined by transmission electron microscopy (TEM); high resolution TEM (HRTEM) was used to provide a close overview of the final composite material, as shown in Fig. 2. The MoS_2_ sheets were located horizontally on the surface of g-C_3_N_4_, which forms intimate interfacial contact at the 2D heterojunction (Fig. 2a). The interaction that formed a heterojunction at the interface may improve the contact area, which is helpful for improving the charge separation process and may enhance the overall photocatalytic activity of MoS_2_-g-C_3_N_4_-1. Figures S2 and S3 presents a TEM image of the BM-g-C_3_N_4_, which shows a thick and agglomerated sheet-like structure. Figure 2c,d,e, and f shows the presence of C, N, Mo, and S in MoS_2_-g-C_3_N_4_-1, which confirms the absence of other impurities. These results clearly show that MoS_2_ and g-C_3_N_4_ are attached closely to form a heterostructure. Energy-filtered transmission electron microscopy with the corresponding element mapping and EDX analysis were also performed to determine the chemical composition of MoS_2_-g-C_3_N_4_-1 (Fig. 2h).

**Figure 3:**
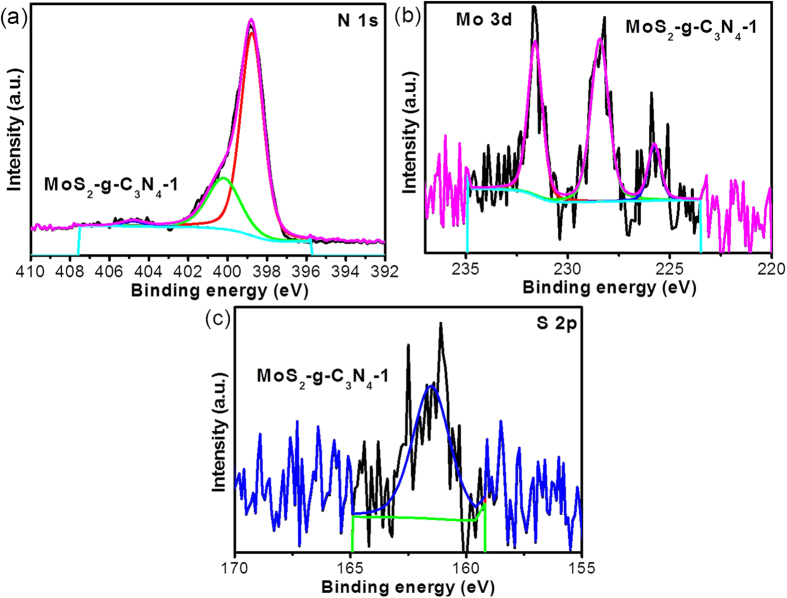
Fitted N 1 s high resolution core level spectra of (**a**) MoS_2_-g-C_3_N_4_-1, (**b**) Fitted Mo 3d, and (**c**) S 2p high resolution core level spectra of MoS_2_-g-C_3_N_4_-1.

The chemical interaction and chemical composition of the AP-g-C_3_N_4_, BM-g-C_3_N_4_, and MoS_2_-g-C_3_N_4_ heterostructure were examined by XPS and the results are presented in Fig. 3. The wide survey spectra of the above samples clearly indicate the presence of C, N, S, and Mo. The two peaks at ~284.53 eV and ~287.99 eV in the C 1 s high resolution spectra of the AP-g-C_3_N_4_, BM-g-C_3_N_4_, and MoS_2_-g-C_3_N_4_ heterostructures (Figure S4) were assigned to carbon-containing contamination and characteristic C-N coordination, respectively, which is generally observed in carbon nitride. Figure S5a,b, and Fig. 3a show the high resolution N 1 s core level spectra with the corresponding deconvoluted Gaussian fitted peaks of AP-g-C_3_N_4_, BM-g-C_3_N_4_, and MoS_2_-g-C_3_N_4_ heterostructures. The N 1 s spectra of carbon nitride is generally ascribed to different types of C-N coordination in the sample according to their respective binding energies (BEs). For example, the peak at BEs of ~398.5, ~400.15 eV, and ~404.5 eV were assigned to the C-N-C, N-(C)_3_, and C-N-H, respectively^16^,^17^. Interestingly, the N 1 s binding energy of the MoS_2_-g-C_3_N_4_ heterostructure shifted slightly toward a higher value compared to the comparative materials, which could be associated with the presence of interfacial electronic interactions between the MoS_2_ and g-C_3_N_4_. These electronic interactions may helpful in enhancing the recombination lifetime of the charge carrier and the photocatalytic activity of the heterostructure.

The high resolution Mo 3d photoelectron spectrum shows two peaks at ~228.41 and ~231.59 eV, which corresponds the doublet of Mo 3d_5/2_ and Mo 3d_3/2_, respectively, (Fig. 3b). Figure 3c presents the peak at ~161.75 and ~162.92 eV for the photoelectron peak of S 2p, which was attributed to the splitting of S 2p_3/2_ and S 2p_1/2_, respectively. On the other hand, the latter weak peak was assigned to C-S coordination in the present heterostructure. This is also consistent with the findings reported elsewhere^16^,^26^. These results clearly show that the MoS_2_ sheet had been deposited on the g-C_3_N_4_ surface during the simultaneous exfoliation process in the ball milling jar.

### Visible Induced Photocatalytic Performance

**Figure 4:**
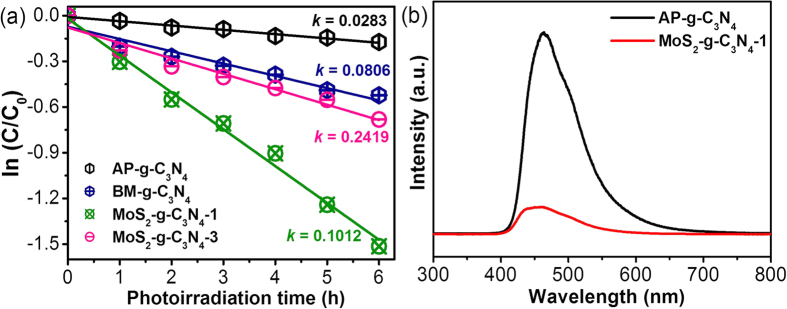
(**a**) Kinetic fit for the degradation of RhB over AP-g-C_3_N_4_, BP-g-C_3_N_4_, MoS_2_-g-C_3_N_4_-1 heterostructure, and MoS_2_-g-C_3_N_4_-3 heterostructure under visible photoirradiation and (**b**) PL spectra of AP-g-C_3_N_4_, and MoS_2_-g-C_3_N_4_-1 heterostructure.

The photocatalytic activity of the MoS_2_-g-C_3_N_4_ heterostructure was evaluated by the degradation of the rhodium blue (RhB) model organic pollutant under visible photoirradiation, which is used frequently in the textile industries. The results were compared with those of the bare materials, i.e., AP-g-C_3_N_4_ and BP-g-C_3_N_4_. The degradation results of RhB showed that AP-g-C_3_N_4_ and BP-g-C_3_N_4_ exhibit poor photocatalytic activity, whereas g-C_3_N_4_ loaded with different MoS_2_ contents showed enhanced photocatalytic activity, which was attributed to the moderate band gap and unique interfacial interaction and electronic structure of MoS_2_ and g-C_3_N_4_. Among them, the MoS_2_-g-C_3_N_4_-1 heterostructure showed better photocatalytic activity than the AP-g-C_3_N_4_, BP-g-C_3_N_4_, and MoS_2_-g-C_3_N_4_-3 heterostructure (Figure S6). To understand the precise degradation rate of the AP-g-C_3_N_4_, BP-g-C_3_N_4_, MoS_2_-g-C_3_N_4_-1 heterostructure, and MoS_2_-g-C_3_N_4_-3 heterostructure, the rate constant (k) was calculated using the equation reported elsewhere^27^,^28^. Figure 4 shows the degradation rate of the AP-g-C_3_N_4_, BP-g-C_3_N_4_, MoS_2_-g-C_3_N_4_-1 heterostructure, and MoS_2_-g-C_3_N_4_-3 heterostructure as a function of the visible photoirradiation times. The apparent *k* for the degradation of RhB was approximately 0.0283/h, 0.0806/h, 0.2419/h, and 0.1012/h for AP-g-C_3_N_4_, BP-g-C_3_N_4_, MoS_2_-g-C_3_N_4_-1 heterostructure, and MoS_2_-g-C_3_N_4_-3 heterostructure, respectively.

These results clearly show that the MoS_2_-g-C_3_N_4_-1 heterostructure with the optimized MoS_2_ loading exhibits a higher degradation rate than the AP-g-C_3_N_4_, BP-g-C_3_N_4_, and MoS_2_-g-C_3_N_4_-3 heterostructure. In addition, the further addition of an excess of MoS_2_ led to a decrease in photocatalytic efficiency due to the following factors. First, an excess of MoS_2_ may poison of some of the active sites of the catalyst because of its two dimensional structure, which hinders the effective generation and separation of photoinduced charge carriers. Second, the larger amount of MoS_2_ can lead to an increase in the opacity of the solution, which prevents light from passing through the reaction solution, resulting in poor photocatalytic activity. In this scenario, a suitable and optimal amount of catalyst is significant for optimizing the photocatalytic performance of the heterostructures^29^.

The enhanced photocatalytic activities of the photocatalyst are generally related to the generation of photogenerated charge carriers. Therefore, for further verification of interfacial charge transfer and separation efficiency of the photoinduced charge carriers over the surface of the photocatalysts, the photoluminescence emission spectra of AP-g-C_3_N_4_ and MoS_2_-g-C_3_N_4_-1 were acquired with an excitation wavelength of 325 nm. The acquired emission intensity in the PL spectra generally reflects the recombination rate of the photogenerated electron/hole pairs. A higher emission intensity indicates rapid charge recombination of the photogenerated charge carriers, whereas a lower luminescence intensity indicates a slow recombination rate^13^,^14^,^16^,^17^. This means that a large number of photogenerated charge carriers are present at the surface of the photocatalyst. This could be used for a range of redox reactions, which are favorable for achieving a higher photocatalytic activity. As shown in Fig. 4b, a strong emission band was observed in the case of AP-g-C_3_N_4_, which is the general sign of a fast recombination rate of charge carriers, whereas this peak was reduced remarkably after the addition of MoS_2_-g-C_3_N_4_-1, which suggests that the recombination efficiency of the photogenerated charge carrier over the heterojunction surface can be prevented effectively by the addition of MoS_2_^13^,^14^,^16^,^26^. Based on the above results, MoS_2_-g-C_3_N_4_-1 might exhibit enhanced photocatalytic activity, which was confirmed by the dye degradation experiments under visible photoirradiation.

**Figure 5:**
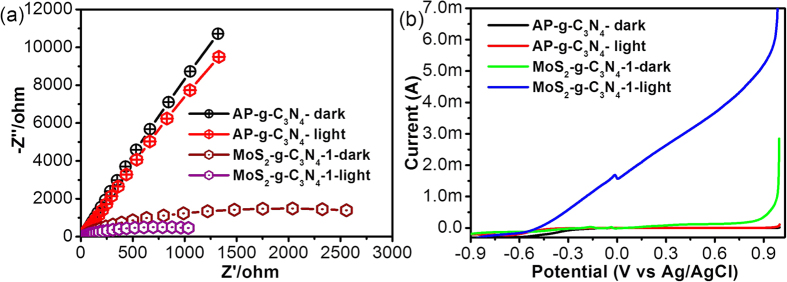
(**a**) Nyquist plot and (**b**) Qualitative LSV voltammogram of AP-g-C_3_N_4_, and MoS_2_-g-C_3_N_4_-1 heterostructure.

EIS of different electrodes were performed in the dark and under visible photoirradiation to gain more insight in the charge transfer phenomenon and photogenerated electrons-holes recombination properties over the surface of the MoS_2_-g-C_3_N_4_-1, as shown in Fig. 5a. Generally, the semicircular arc in the EIS spectra demonstrates the transportation, effective separation, and electron transfer resistance of the photogenerated electron-hole pairs occurring over the electrode surface. The reflectance of the smallest semicircular diameter in the EIS plot showed a smaller charge transfer resistance, effective photo generated electron-hole pair separation, and faster interfacial charge transfer, whereas the largest semicircular diameter reflected the large charge transfer resistance, high recombination rate of the photo generated electron-hole pairs and poor interfacial charge transfer at the electrode surface^3^,^10^,^13^,^16^,^21^,^24^. In light of the above discussion, the results clearly show that the MoS_2_-g-C_3_N_4_-1 electrode under visible photoirradiation has a lower charge transfer resistance than the g-C_3_N_4_ electrode, which indicates the effective photo generated electron-hole pair separation and faster interfacial charge transfer at the MoS_2_-g-C_3_N_4_-1 surface compared to the bare material. These results clearly show that the resistance (charge transfer/interfacial) over the surface of the bare electrode was reduced significant by the addition of MoS_2_. These results also revealed an analogous trend with the photocatalytic activity.

The enhanced photocatalytic performance of the MoS_2_-g-C_3_N_4_-1 heterostructure was confirmed by linear sweep voltammetry (LSV) qualitative photoelectrochemical measurements in the dark and under visible photoirradiation^3^,^10^. Figure 5b shows that the photocurrent response of the MoS_2_-g-C_3_N_4_-1 heterostructure under visible photoirradiation was much higher than that of the bare material. This may be due to the addition of MoS_2_, which may enhance the recombination lifetime of the charge carriers under visible light irradiation. These results further support the effective formation of a heterojunction between MoS_2_ and g-C_3_N_4_, which helps improve the effective separation of photogenerated electron and holes, thereby enhancing the photo response of the heterostructure compared to the bare material. These results are also in accordance with the degradation results under similar photoirradiation conditions.

**Figure 6:**
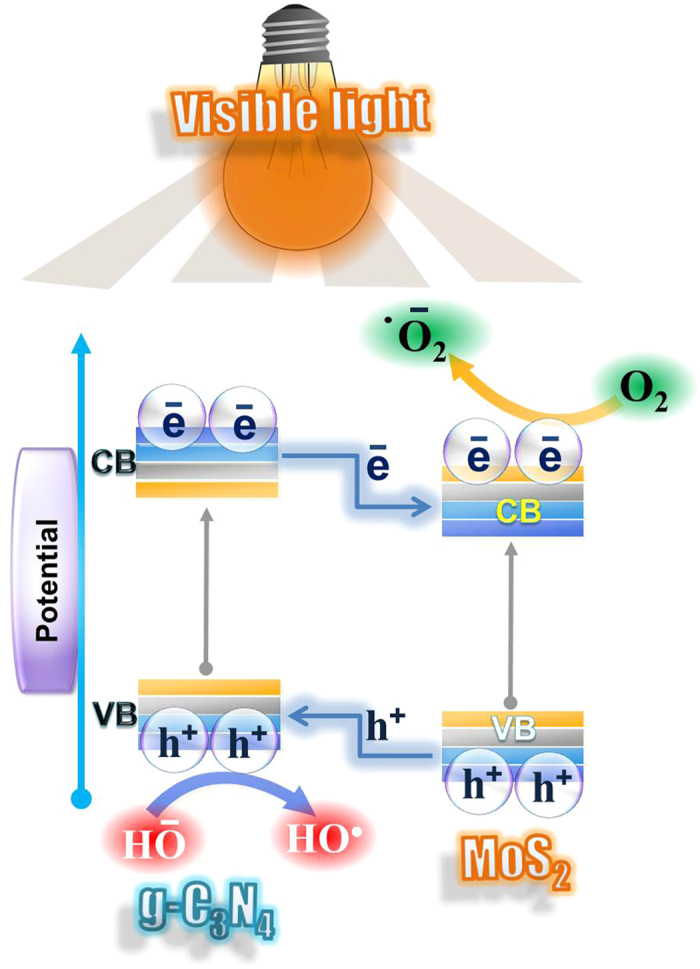
Proposed mechanism for the possible charge transfer movement and separation process occurred under visible photoirradiation over the MoS_2_-g-C_3_N_4_-1 heterostructure interface.

To shed more light on the enhancement of the photocatalytic activity of the MoS_2_-g-C_3_N_4_-1, the band edge potential position of the MoS_2_ and g-C_3_N_4_ was studied to gain more insight into the migration and flow of photogenerated electrons and holes at the heterojunction surface. The band gap of the g-C_3_N_4_ and MoS_2_ obtained from the UV-visible absorption spectra was 2.78 eV and 1.09 eV, respectively, which are also accordance with previous reports^24^,^26^. Using the estimated band gap, the band-edge potential position of the conduction band and valence band were evaluated using the following empirical formula^26^:


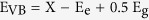



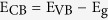


where E_VB_ and E_CB_ are the valence and conduction band-edge potential positions, respectively. X is the absolute electronegativity of the semiconductor materials. E_e_ is the estimated energy of the free electrons on the hydrogen scale, which is approximately 4.5 eV. E_g_ is the band gap mobility of the semiconductor materials. Based on the above equations, the derived VB and CB band edge potential position of MoS_2_ were 1.81 eV and −0.09 eV, respectively, whereas they were 1.65 eV and −1.20 eV for g-C_3_N_4_. These edge potentials are favorable for the migration of photogenerated charge carriers through the material interface. Under visible photoirradiation, both photocatalysts were excited because of their narrow band gap energy and generated the corresponding electron and holes (Fig. 6)^30^. The excited photogenerated CB electron of g-C_3_N_4_ jumps easily to the CB of MoS_2_ via the heterojunction owing to its more negative band edge potential. Simultaneously, the photogenerated holes formed in the VB of MoS_2_ can migrate to the VB of g-C_3_N_4_. As a result, the simultaneous jumping and migration of photogenerated electrons and holes through the heterojunction interfaces promotes the effective separation of photogenerated electron and holes by reducing the likelihood of photogenerated electron/hole recombination and provide an excess of the electrons in the CB of MoS_2_ and holes in the VB of g-C_3_N_4_. These available photogenerated electrons and holes in MoS_2_-g-C_3_N_4_-1 promoted the oxidative and reductive reactions for the degradation of the pollutant under visible light irradiation. The photogenerated and excited electrons collected on the surface of the nanohybrid were then trapped by the dissolved oxygen molecules in water to yield the superoxide radical anions (•O_2_^−^), whereas the holes located on the surface could react with the surface adsorbed hydroxyl ions to form highly reactive HO^•^. These highly reactive radicals are responsible for the photodegradation and mineralization of pollutants^31^,^32^,^33^,^34^.

In addition to these results, the specific surface areas of the AP-g-C_3_N_4_, BP-g-C_3_N_4_, and MoS_2_-g-C_3_N_4_-1 heterostructure were examined because the surface plays an important role in enhancing the catalytic activity of the material by providing sufficient contact between the pollutant or dyes and the surface of the photocatalyst. The Brunauer-Emmett-Teller (BET) specific surface area of the MoS_2_-g-C_3_N_4_-1 heterostructure was 6.40 m^2^/g, which is higher than that of AP-g-C_3_N_4_ (3.4846 m^2^/g) and BP-g-C_3_N_4_ (3.5468 m^2^/g). The BJH Adsorption and desorption cumulative surface area of pores of MoS_2_-g-C_3_N_4_-1 heterostructure was 5.5541 m^2^/g and 6.8061 m^2^/g whereas BJH Adsorption and desorption cumulative surface area of pores of AP-g-C_3_N_4_ was 1.070 m^2^/g 1.971 m^2^/g.

### Half-Cell Electrochemical Performance

The half-cell electrochemical performance of the MoS_2_-g-C_3_N_4_-1 along with their bare materials were examined by cyclic voltammetry (CV) and galvanostatic charge-discharge (GCD), which are used widely to assess the electrochemical supercapacitance of the as-synthesized materials. The electrochemical performance of the representative heterostructure was compared with that of the ball milled g-C_3_N_4_ because most of the properties are similar to those of the as- prepared g-C_3_N_4_.

**Figure 7:**
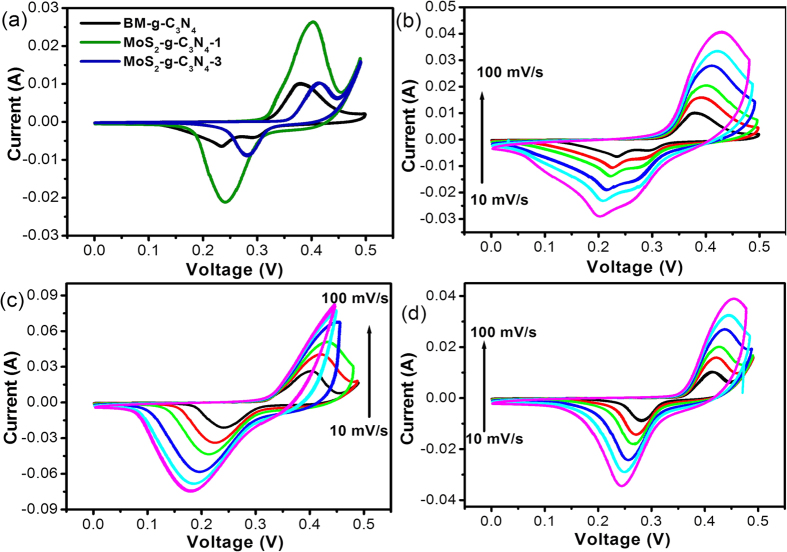
(**a**) Comparative cyclic voltammogram of the BP-g-C_3_N_4_, MoS_2_-g-C_3_N_4_-1, and MoS_2_-g-C_3_N_4_-3 heterostructure at a scan rate of 10 mV s^−1^, (**b**) cyclic voltammogram of BM-g-C_3_N_4_ at a scan rate of 10–100 mV s^−1^, (**c**) cyclic voltammogram of MoS_2_-g-C_3_N_4_-1 at a scan rate of 10–100 mV s^−1^, and (**d**) cyclic voltammogram of MoS_2_-g-C_3_N_4_-3 at a scan rate of 10–100 mV s^−1^.

Figure 7 shows the comparative CV profile of the AP-g-C_3_N_4_, BM-g-C_3_N_4_, MoS_2_-g-C_3_N_4_-1, and MoS_2_-g-C_3_N_4_-3 heterostructure, in which the MoS_2_-g-C_3_N_4_-1 heterostructure electrode exhibits stronger electrochemical supercapacitive behavior with a large capacitive area compared to the bare material. This improved performance of the g-C_3_N_4_ after the addition of MoS_2_ might be due to the intimate interfacial interaction between the g-C_3_N_4_ and MoS_2_, which provides the effective migration of charge carriers from the MoS_2_ layers edges to g-C_3_N_4_^35^,^36^. The CV profile of the BM-g-C_3_N_4_, MoS_2_-g-C_3_N_4_-1, and MoS_2_-g-C_3_N_4_-3 heterostructure was also measured at different scan rates and the results are presented in Fig. 7b,c and d. The current increased with increasing scan rate and MoS_2_-g-C_3_N_4_-1 exhibited a large capacitive area with superior capacitance behavior than the other bare materials.

**Figure 8:**
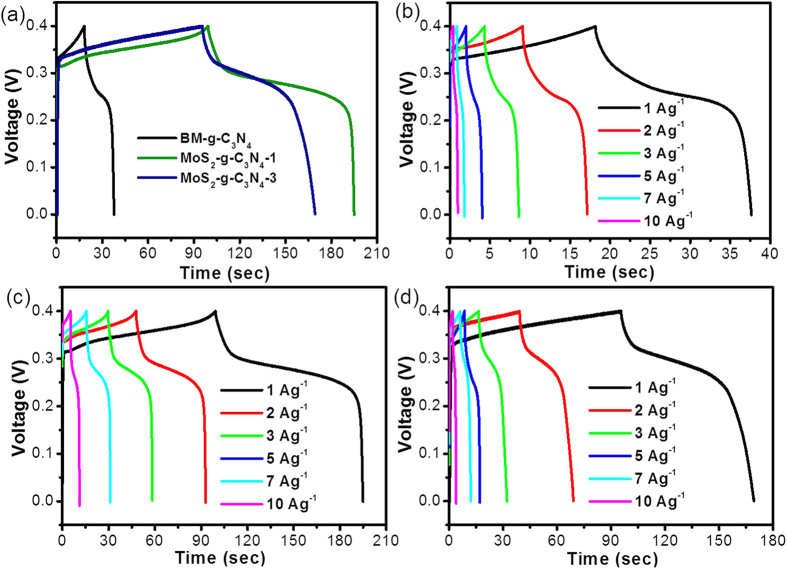
(**a**) Comparative galvanostatic CD profile of the BP-g-C_3_N_4_, MoS_2_-g-C_3_N_4_-1, and MoS_2_-g-C_3_N_4_-3 heterostructure electrode at a current load of 1 Ag^−1^, (**b**) Galvanostatic CD curves of BP-g-C_3_N_4_ at a current load of 1–10 Ag^−1^, (**c**) Galvanostatic CD curves of MoS_2_-g-C_3_N_4_-1 at a current load of 1–10 Ag^−1^, and (**d**) Galvanostatic CD curves of the MoS_2_-g-C_3_N_4_-3 heterostructure at a current load of 1–10 Ag^−1^.

To obtain more information on the rapid potential drop and slow potential decay behavior of MoS_2_-g-C_3_N_4_-1 compared to the bare material, galvanostatic charge-discharge experiments were performed at different current densities and the corresponding specific capacitance was calculated using the reported formula. Figure 8a presents the comparative GCD profile of the AP-g-C_3_N_4_, BM-g-C_3_N_4_, and MoS_2_-g-C_3_N_4_-1 heterostructure for a better understanding, in which the MoS_2_-g-C_3_N_4_-1 heterostructure showed a higher specific capacitance (240.85 F/g) compared to the BM-g-C_3_N_4_ (48.77 F/g) and MoS_2_-g-C_3_N_4_-3 heterostructure (185.7 F/g) at 1 Ag^−1^. The galvanostatic charge and discharge behavior of BM-g-C_3_N_4_, MoS_2_-g-C_3_N_4_-1, and MoS_2_-g-C_3_N_4_-3 heterostructure was also examined at different current densities; Fig. 8b,c and d show the resulting profile. In addition, Fig. 8c shows that MoS_2_-g-C_3_N_4_-1 displayed a short charging time and longer discharging time than the bare materials, which also indicate the capacitance of MoS_2_-g-C_3_N_4_-1. The heterostructure of g-C_3_N_4_ with a low MoS_2_ content exhibited improved capacitance behavior compared to the pure g-C_3_N_4_, whereas the further addition of MoS_2_ led to a decrease in capacitance. This may be due to the restacking of MoS_2_ in the heterostructure, which will reduce the active sites and decrease the overall performance of the heterostructures^37^.

The specific capacitance of BM-g-C_3_N_4_ obtained at 1, 2, 3, 5, 7, and 10 Ag^−1^ current loads were 48.77, 40.75, 32.32, 24.75, 15.92, and 15.25 F g^−1^, respectively. Similarly, a specific capacitance of 240.85, 227.5, 215.02, 183.37, 182.7, and 147.75 F g^−1^ were found for MoS_2_-g-C_3_N_4_-1 at current loads of 1, 2, 3, 5, 7, and 10 Ag^−1^, respectively. In contrast, 185.7, 160, 122.32, 106.62, 98.7, and 47.75 F g^−1^ were obtained for MoS_2_-g-C_3_N_4_-3 at current loads of 1, 2, 3, 5, 7, and 10 Ag^−1^, respectively. These specific capacitance measurements revealed MoS_2_-g-C_3_N_4_-1 to have a higher capacitance than the BM-g-C_3_N_4_ and MoS_2_-g-C_3_N_4_-3 heterostructure that still remained high at a higher current density.

The cyclic stability test of MoS_2_-g-C_3_N_4_-1 was also conducted to explore the possibility of the long term performance of the electrodes by repeating the charge-discharge cycling test at a current load of 5 Ag^−1^. The cyclic stability results revealed the impressive capacitance retention of the MoS_2_-g-C_3_N_4_-1 heterostructure electrode, which further highlights its good cyclic stability as an electrode material (Figure S7). This enhanced capacitance of MoS_2_-g-C_3_N_4_-1 can be attributed mainly to the high surface area generated by high energy ball milling and the intimate interfacial interaction. This most likely reduces the diffusion path of ions, thereby allowing the rapid transportation of electrons between the electrodes and electrolyte, which enhances the overall capacitance of the materials.

## Conclusions

A facile, inexpensive, and sustainable synthesis strategy was used to synthesize MoS_2_-g-C_3_N_4_ heterostructures and study its visible responsive photocatalytic activity and electrochemical half-cell performance. The MoS_2_-g-C_3_N_4_ heterostructure with the optimal MoS_2_ content showed significantly enhanced photodegradation ability under visible photoirradiation and electrochemical supercapacitive performance compared to AP-g-C_3_N_4_ and BP-g-C_3_N_4_. The optimized MoS_2_-g-C_3_N_4_ heterostructure exhibited approximately 9 times higher photodegradation ability than the bare material. In addition, the MoS_2_-g-C_3_N_4_ heterostructure also exhibited higher capacitance than BP-g-C_3_N_4_, which is due to the unique design and intimate contact between the starting materials. The improved performance was attributed to the intimate interfacial interaction between the MoS_2_ and g-C_3_N_4_, suitable band gap, effective separation of photogenerated charge carriers, high surface area, and nitrogen content. This study shows that simple mechanical milling is an inexpensive method for preparing large amounts of a material with high performance for environmental and energy storage applications. Overall, this method is an efficient and energy saving technology that can easily induces various types of reactions and material characteristics.

## Experimental Section

### Materials

The commercially available model organic pollutant, RhB, as well as molybdenum sulfide, and nafion resin solution, were purchased from Sigma-Aldrich. Potassium chloride (KCl) and sodium sulfate (Na_2_SO_4_) were obtained from Duksan Pure Chemicals Co. Ltd. South Korea. Ethyl cellulose and α-terpineol were supplied by KANTO Chemical Co., Japan. The melamine was acquired from Duksan. Nickel foam was purchased from MTI Corporation, USA (thickness 1.6 mm, surface density 346 g m^−2^ and porosity ≥95%) and the fluorine-doped transparent conducting oxide glass used for photoelectrode preparation (FTO; F-doped SnO_2_ glass; 7 Ω/sq) was obtained from Pilkington, USA. All other chemicals and deionized water (obtained from a PURE ROUP 30 water purification system) used in this study were of analytical grade and used as received.

### Methods

X-ray diffraction (XRD PANalytical, X’pert PRO-MPD, Netherland) was performed using Cu Kα radiation (λ = 0.15405 nm). The optical properties of the photocatalyst were examined by ultraviolet-visible-near infrared spectrophotometry (UV-VIS-NIR, Cary 5000, VARIAN, USA). The chemical interaction and surface behavior of the as-synthesized materials were studied by X-ray photoelectron spectroscopy (XPS, ESCALAB 250 XPS, Thermo Fisher Scientific U.K.) using monochromatized Al Kα X-rays (hν = 1486.6 eV). The photoluminescence spectroscopy (PL, Kimon, 1 K, Japan) was performed over the scanning range, 200–800 nm. The excitation wavelength was 325 nm and this experiment was conducted at the Korea Basic Science Institute, Gwangju Center, South Korea. The internal structure of the optimized photocatalyst was performed by field emission transmission electron microscopy (FE-TEM, Tecnai G2 F20, FEI, USA) operated at a 200 kV accelerating voltage. An accelerated surface area and porosimetry system (ASAP 2020, Physisorption Analyzer Micromeritics Inc. USA) was used to examine the surface area of the samples with the help of N_2_ adsorption-desorption isotherms and the Brunauer-Emmett Teller (BET) method. The light source used for visible photoirradiation in the photocatalytic and photoelectrochemical experiments was acquired from 3 M USA. The intensity and wavelength of the lamp was 31 mW/cm^2^ and >500 nm, respectively. The VersaSTAT 3 used to measure the electrochemical supercapacitance and photoelectrochemical performance of the as-synthesized materials was acquired from Princeton Research USA and was equipped with a three electrode assembly cell system. Ag/AgCl (3.0 M KCl) and platinum sheet were used as the reference and counter electrodes, respectively.

### Model pollutant degradation test

The photocatalytic ability of the as-synthesized samples to degrade the model organic pollutant, RhB dye, was tested under visible light photodegradation. A 250 mg/L sample of the photocatalyst was dispersed in aqueous 5 g/L RhB solution. Prior to visible photoirradiation, the resulting dispersion was stirred magnetically for 30 min in the dark to determine the adsorption-desorption equilibrium. After the desired photoirradiation time, a 2 mL sample was collected and the catalyst was removed by centrifugation to obtain a clear liquid. The absorption spectra of the above clear solution were recorded on a UV–vis spectrophotometer and the degradation efficiency was calculated using the absorption spectra.

### Photoelectrode fabrication and its visible light-driven photoelectrochemical studies

For the photoelectrochemical test, the photoelectrode was fabricated using a method similar to that reported elsewhere. Briefly, a paste of the catalyst was prepared by mixing the as-prepared photocatalyst in ethyl cellulose and α-terpineol through proper mixing. The resulting paste was coated on the FTO glass electrode using the doctor blade method with an effective area of 1 cm^2^. The coated electrodes were dried using a drying lamp and used for the EIS and LSV measurements in an aqueous Na_2_SO_4_ electrolyte. EIS and LSV analysis was performed on an electrochemical work station and the Nyquist plots were recorded over the 1 to 10^4^ Hz frequency range. The photocurrent was recorded at a scan rate of 50 mV/s over a potential range, −0.9 to +0.9 V, in the dark and under visible photoirradiation.

### Electrode fabrication and its electrochemical capacitance measurements

The MoS_2_-g-C_3_N_4_ working electrodes were fabricated by coating the slurry of the active materials (MoS_2_-g-C_3_N_4_ heterostructure and BM-g-C_3_N_4_) on the nickel foam^10^. The slurry was prepared by dispersing the active material with carbon black and nafion in an ethanol solution, sonicated for 5 min, and coated on the commercial available nickel foam. The active material-coated foam was dried under a lamp and used as the working electrode. The initial electrochemical behavior of the electrodes was examined by CV at different scan rates. The charge discharge curve was obtained from the galvanostatic CD measurements and the specific capacitance was calculated using the equation reported elsewhere.

### Mechanical ball milling of commercial MoS_2_ and g-C_3_N_4_

#### Preparation of AP-g-C_3_N_4_

The previous well-reported heating method of melamine was performed to synthesize g-C_3_N_4_^1^,^2^,^10^. Briefly, a quartz container containing 5 g of melamine was placed inside the furnace at 500 °C for 2 h under a constant N_2_ flow. After the reaction was complete, light yellow colored samples were collected and ground to a powder using a mortar and pestle.

#### Preparation of MoS_2_-g-C_3_N_4_

The MoS_2_-g-C_3_N_4_ heterostructure with different weight percentages of MoS_2_ were prepared by mechanically grinding MoS_2_ and g-C_3_N_4_ in a ball milling jar. The MoS_2_-g-C_3_N_4_ heterostructure containing 10% of MoS_2_ (MoS_2_-g-C_3_N_4_-1) and 30% (MoS_2_-g-C_3_N_4_-3) was ground mechanically for 12 h at a fixed rotation per minute (400 rpm). The as-synthesized g-C_3_N_4_ (AP-g-C_3_N_4_), which is the matrix material of the MoS_2_-g-C_3_N_4_ heterostructure, was also milled under similar conditions and is abbreviated as BM-g-C_3_N_4_. The above synthesis process clearly shows the importance and simplicity of the present method for bulk production of the material, which may be helpful for future applications.

## Additional Information

**How to cite this article:** Ansari, S. A. and Cho, M. H. Simple and Large Scale Construction of MoS_2_-g-C_3_N_4_ Heterostructures Using Mechanochemistry for High Performance Electrochemical Supercapacitor and Visible Light Photocatalytic Applications. *Sci. Rep.* **7**, 43055; doi: 10.1038/srep43055 (2017).

**Publisher's note:** Springer Nature remains neutral with regard to jurisdictional claims in published maps and institutional affiliations.

## Supplementary Material

Supporting Information
