# Supplementary material for: Simple and Large Scale Construction of MoS2-g-C3N4 Heterostructures Using Mechanochemistry for High Performance Electrochemical Supercapacitor and Visible Light Photocatalytic Applications
Source: Sci Rep. 2017 Feb 27;7:43055. doi: 10.1038/srep43055 (PMC5327403; doi:10.1038/srep43055)
Supplement: Supporting Information [file srep43055-s1.pdf]

Supporting Information

Simple and Large Scale Construction of MoS_2-g-C_3N_4 Heterostructures Using Mechanochemistry for High Performance Electrochemical Supercapacitor and Visible Light Photocatalytic Applications

Sajid Ali Ansari[1*] and Moo Hwan Cho[1*]

[1]School of Chemical Engineering, Yeungnam University, Gyeongsan-si, Gyeongbuk 712-749, South Korea, Phone: +82-53-810-2517; Fax: +82-53- 810-4631.

[*]Corresponding authors: mhcho@ynu.ac.kr, sajidansari@ynu.ac.kr

UV-vis Diffuse absorption spectra of the AP-g-C_3N_4, BM-g-C_3N_4, P-MoS_2, MoS_2-g-C_3N_4-1, and MoS_2-g-C_3N_4-3 heterostructure

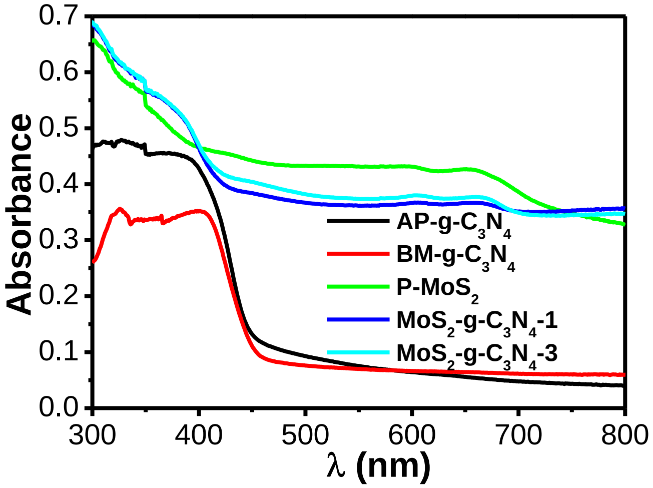

Figure S1. UV-vis Diffuse absorption spectra of the AP-g-C_3N_4, BM-g-C_3N_4, P-MoS_2, MoS_2-g-C_3N_4-1, and MoS_2-g-C_3N_4-2 heterostructure.

TEM Image of the BM-g-C_3N_4

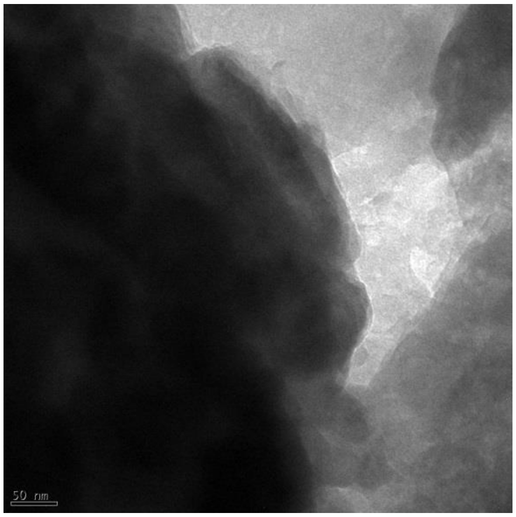

Figure S2. TEM image of the BM-g-C_3N_4.

TEM Image of the BM-g-C_3N_4

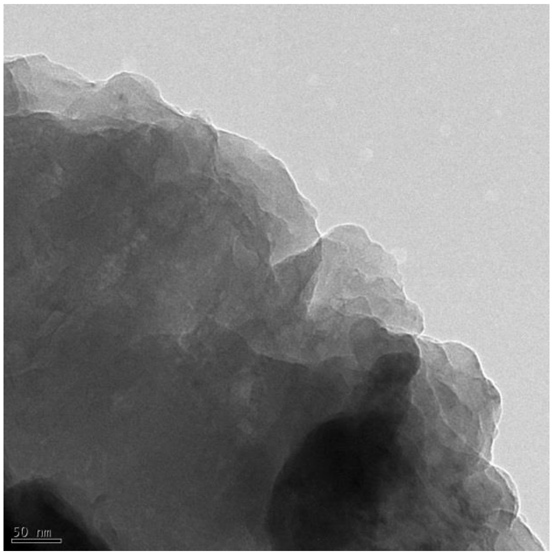

Figure S3. TEM image of the BM-g-C_3N_4.

High resolution C 1s core level spectra of AP-g-C_3N_4, BM-g-C_3N_4, and MoS_2-g-C_3N_4-1

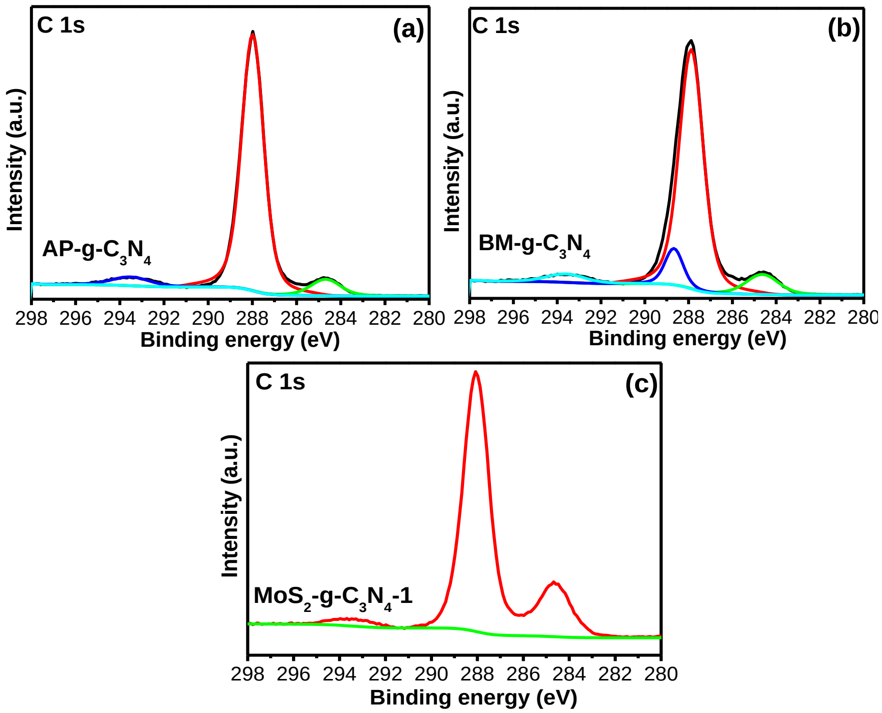

Figure S4. High resolution C 1s core level spectra of (**a**) AP-g-C_3N_4, (**b**) BM-g-C_3N_4, (**c**) MoS_2-g-C_3N_4-1 heterostructure.

High resolution N 1s core level spectra of AP-g-C_3N_4, BM-g-C_3N_4

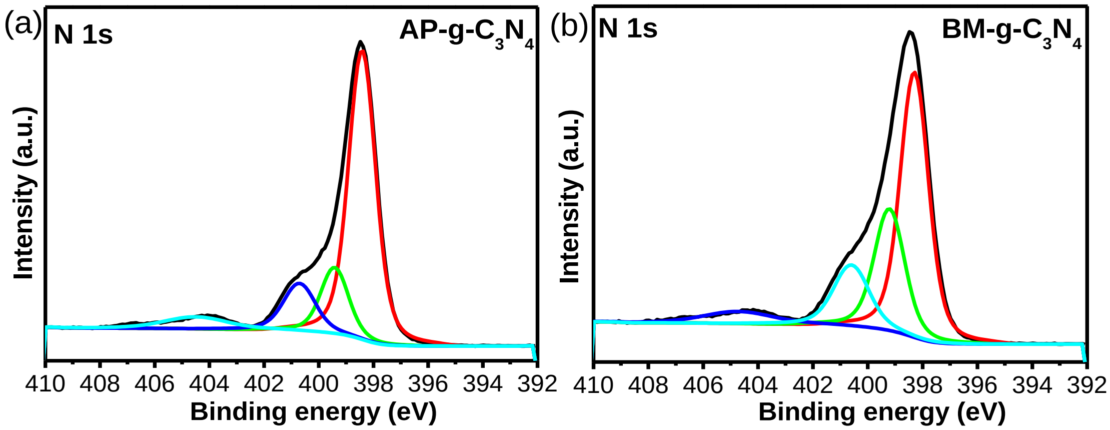

Figure S5. Fitted N 1s high resolution core level spectra of (**a**) AP-g-C_3N_4, (**b**) BM-g-C_3N_4.

Photocatalytic activity of the RhB degradation as a function of the visible light photoirradiation over AP-g-C_3N_4, BP-g-C_3N_4, MoS_2-g-C_3N_4-1 heterostructure, and MoS_2-g-C_3N_4-3 heterostructure under visible photoirradiation

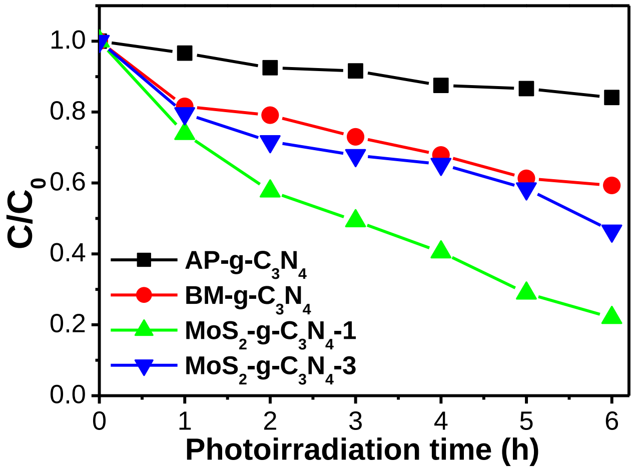

Figure S6. Photocatalytic activity of the RhB degradation as a function of the visible light photoirradiation over AP-g-C_3N_4, BP-g-C_3N_4, MoS_2-g-C_3N_4-1 heterostructure, and MoS_2-g-C_3N_4-3 heterostructure under visible photoirradiation.

Cyclic stability test

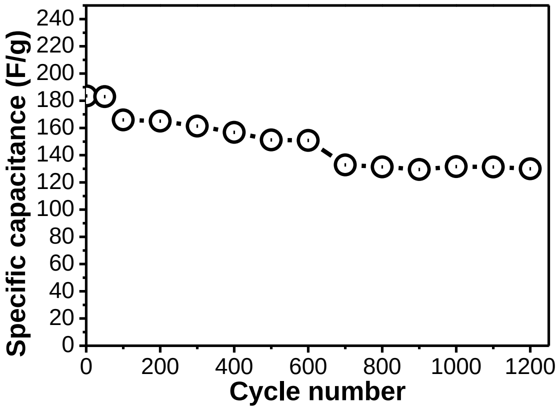

Figure S7. Cyclic stability performance of the MoS_2-g-C_3N_4-1 heterostructure electrode at a constant current load of 5 A/g.
